# The fitness effects of delayed switching to sex in a facultatively asexual insect

**DOI:** 10.1002/ece3.3895

**Published:** 2018-02-06

**Authors:** Nathan W. Burke, Russell Bonduriansky

**Affiliations:** ^1^ Evolution & Ecology Research Centre School of Biological, Earth and Environmental Sciences University of New South Wales Australia Sydney NSW Australia

**Keywords:** facultative parthenogenesis, maintenance of sex, Phasmatodea, reproductive modes, reproductive switching, sexual conflict

## Abstract

Facultative reproductive strategies that incorporate both sexual and parthenogenetic reproduction should be optimal, yet are rarely observed in animals. Resolving this paradox requires an understanding of the economics of facultative asexuality. Recent work suggests that switching from parthenogenesis to sex can be costly and that females can resist mating to avoid switching. However, it remains unclear whether these costs and resistance behaviors are dependent on female age. We addressed these questions in the Cyclone Larry stick insect, *Sipyloidea larryi*, by pairing females with males (or with females as a control) in early life prior to the start of parthenogenetic reproduction, or in mid‐ or late life after a period of parthenogenetic oviposition. Young females were receptive to mating even though mating in early life caused reduced fecundity. Female resistance to mating increased with age, but reproductive switching in mid‐ or late life did not negatively affect female survival or offspring performance. Overall, mating enhanced female fitness because fertilized eggs had higher hatching success and resulted in more adult offspring than parthenogenetic eggs. However, female fecundity and offspring viability were also enhanced in females paired with other females, suggesting a socially mediated maternal effect. Our results provide little evidence that switching from parthenogenesis to sex at any age is costly for *S. larryi* females. However, age‐dependent effects of switching on some fitness components and female resistance behaviors suggest the possibility of context‐dependent effects that may only be apparent in natural populations.

## 1 INTRODUCTION

Facultative asexuality—where both sexual and asexual (parthenogenetic) reproduction can occur within the same individual—should be optimal because it combines the benefits of sex and parthenogenesis with fewer of the associated costs (D'Souza & Michiels, 2010). Asexual reproduction provides reproductive assurance when mates are limited (Gerritsen, 1980), and facilitates rapid population growth in new or marginal habitats (Lynch, 1984; Suomalainen, Saura, & Lokki, 1987). Sexual recombination, on the other hand, creates advantageous allele combinations (Kondrashov, 1984), enhances purifying selection (Wagner & Gabriel, 1990), and promotes adaptability in fluctuating environments (Becks & Agrawal, 2012; Burt, 2000). These combined benefits should, in principle, promote the spread of facultative asexuality and the extinction of obligate strategies. Yet, paradoxically, obligate sex is the dominant form of reproduction in animals.

The incidence of facultative asexuality in animals could reflect the costs and benefits of reproductive switching. Switching occurs when facultatively asexual females already producing parthenogenetic offspring from unfertilized eggs change to sexual reproduction when eggs are fertilized. Switching in the reverse direction (from sex to parthenogenesis) may be possible in principle if facultatively parthenogenetic females become sperm limited (e.g., Chang, Ting, Chang, Fang, & Chang, 2014) or if eggs develop resistance to fertilization (e.g., Yashiro & Matsuura, 2014). However, since sperm limitation is unlikely in internally fertilized taxa that store sperm after copulation (such as insects), the incidence of switching back to parthenogenesis after mating is likely to be rare (e.g., Arbuthnott, Crespi, & Schwander, 2015) and may only be possible in very late life (e.g., Chang et al., 2014). For these reasons, we focus predominantly on the reproductive switching that occurs when females reproducing parthenogenetically mate and produce offspring sexually.

Several empirical studies have compared sexual and asexual reproductive performance in internally fertilized facultative systems (e.g., Chang et al., 2014; Corley & Moore, 1999; Matsuura & Kobayashi, 2007). However, explicit assessments of the costs and benefits of switching to sex are scant. A recent study on the facultatively parthenogenetic spiny leaf stick insect, *Extatosoma tiaratum*, found that females that switch from asexual to sexual reproduction suffer significant declines in egg production and lifespan compared to females that reproduce only sexually or only asexually (Burke, Crean, & Bonduriansky, 2015). This suggests that costs of reproductive switching could select against facultative parthenogenesis. However, almost nothing is known about costs of reproductive switching in other facultative organisms.

The magnitude of the cost or benefit to female fitness of switching to sex could depend on the timing of the switch. Parthenogenesis might be most advantageous for facultative asexuals in early life when individual condition is highest, whereas sex might be more beneficial later in life as condition deteriorates with age. This kind of condition‐dependent sex is common in a number of cyclically sexual taxa where stress due to hunger, parasitism, or predator pressure triggers generational switches from asexual to sexual reproduction (Kleiven, Larsson, & Hobek, 1992; Morran, Cappy, Anderson, & Phillips, 2009; West, Gemmill, Graham, Viney, & Read, 2001). Whether aging can trigger a similar condition‐dependent response in facultatively parthenogenetic individuals is not known. Conversely, switching to sex earlier could be more advantageous if costs of sex increase with age—for example, if mating elevates mortality rates in females that switch later in life (see Ram & Hadany, 2016). Likewise, if older females have committed their eggs to a parthenogenetic strategy, mating and fertilization at older ages might interfere with the proper development of these eggs. Finally, females that switch at a young age may benefit by obtaining fitness‐enhancing male factors sooner (see Neiman, 2004), or, if parthenogenetic development is inhibited by physiological or genetic constraints that reduce fecundity or viability (Corley, Blankenship, Moore, & Moore, 1999; Engelstadter, 2008; Vrijenhoek, 1989), by producing a greater proportion of offspring sexually. However, if sexual performance declines relative to asexual performance, then switching in the reverse direction (from sex to parthenogenesis) could become advantageous.

If switching to sexual reproduction is costly for females, it could be subject to sexual conflict, which arises when one sex optimizes its own fitness at the expense of the other (Parker, 1979). Several recent studies suggest that sexual conflict could be an important factor in facultatively asexual organisms (Burke & Bonduriansky, 2017a,b; Burke et al., 2015; Gerber & Kokko, 2016; Kawatsu, 2013a,b, 2015). However, it is unclear what role sexual conflict over timing of mating plays in facultative systems. As occurs in obligately sexual systems, mating at certain ages could be costly for facultatively asexual females, even though males may benefit by mating with females of any age (see Arnqvist & Rowe, 2005). Age‐dependent conflict over mating could therefore mediate selection on facultative parthenogenesis, but this possibility remains unexplored.

Facultatively asexual females are predicted to avoid or resist males whenever mating (or switching) is costly. Females of at least one facultatively asexual species, the spiny leaf insect *Extatosoma tiaratum*, appear to be able to limit costs of switching by resisting mating attempts (Burke et al., 2015). Although age‐dependent costs of switching are expected to correspond to age‐dependent levels of resistance, the extent to which resistance behaviors are age‐dependent in facultatively asexual systems is unclear.

**Figure 1:**
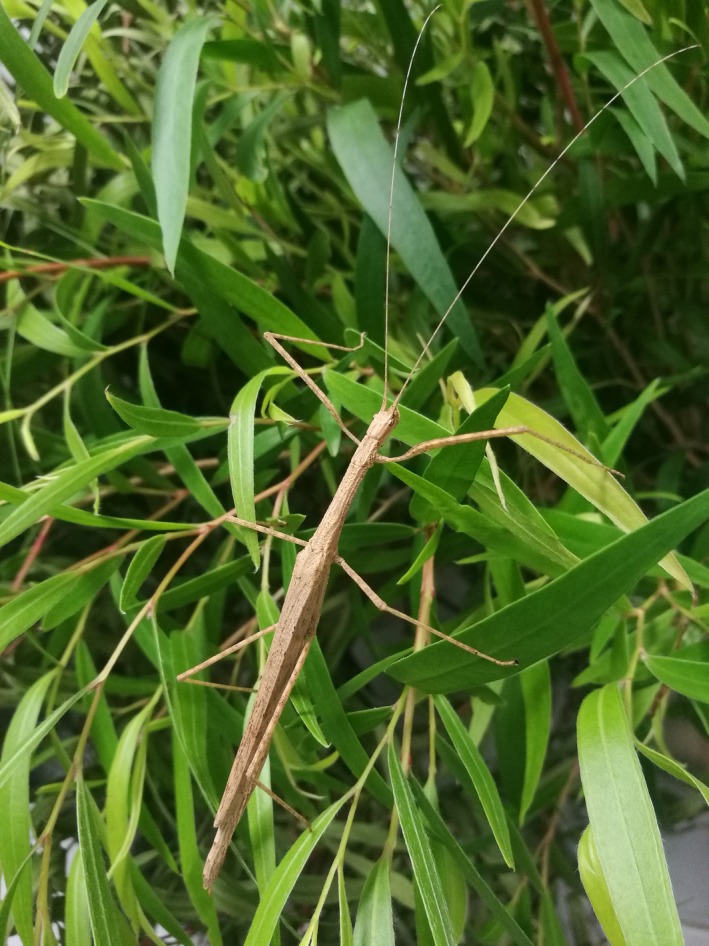
Female Cyclone Larry stick insect, *Sipyloidea larryi*

The Cyclone Larry stick insect, *Sipyloidea larryi* (Figure 1), is a facultatively parthenogenetic phasmatid native to northern Queensland, Australia (Brock & Hasenpusch, 2007, 2009). It feeds on understorey rainforest plants in the wild and is mostly active at night (Brock & Hasenpusch, 2007). Little else is known about the natural history of this species in the wild. Like other facultatively parthenogenetic phasmatids, unfertilized eggs of *S. larryi* hatch into females, whereas fertilized eggs are equally likely to yield sons and daughters, exhibiting a sexual generation time of approximately 1 year (NWB, pers. obs.). Phasmatids such as *S. larryi* are ideal for investigating age‐dependent costs of sex and reproductive switching because they can be experimentally induced to reproduce solely parthenogenetically (by withholding female access to males), solely sexually (by allowing mating and fertilization prior to the onset of parthenogenetic oviposition), or both (by inducing a switch to sex at different reproductive ages following a period of parthenogenesis). Thus, it is possible to compare the economics of switching versus not switching across age classes within a single population. Here, we investigate female mating behavior and reproductive performance in *S. larryi* to test for costs of sex and age‐dependent effects of reproductive switching.

## 2 MATERIALS AND METHODS

### 2.1 Animal maintenance

*Sipyloidea larryi* eggs were obtained from professional insect breeders in New South Wales and Victoria, Australia. Eggs were pooled by breeder origin and hatched in 1‐L cylindrical containers (12 cm diameter × 12 cm high) that held a shallow layer of damp coco‐peat which was moistened with water once a week. Young nymphs were housed communally in 90‐L plastic tubs (38 × 46 × 65 cm) and sexed at the fourth instar. Sexed females were kept individually in cylindrical enclosures (20 cm diameter × 40 cm high) and entered the experiment as focal individuals at adult eclosion. Males were housed communally with other mature males of the same breeder origin that molted in the same week. All nymphs and adults were fed *Agonis flexuosa* leaves, which were replaced weekly and sprayed regularly with water for insects to drink. All eggs, stocks, and experimental animals were raised in a glasshouse with ambient temperature fluctuations from 18 to 27°C.

### 2.2 Experimental design

We manipulated age at pairing (“early‐,” “mid‐” and “late‐life”) and sex of partner (“male‐paired” or “female‐paired”) in a fully factorial experimental design. In the male‐paired treatment, focal females were paired with a single male for three consecutive days at one of three reproductive ages: a week after the last molt when focal females had reached the adult stage but had not yet started to oviposit (early‐life); 14 days following the onset of oviposition (mid‐life); or 28 days following the onset of oviposition (late‐life). These age categories were chosen based on the average reproductive lifespan of females of this species (mean ± *SE*: 41.5 ± 1.4 days). All females were observed to mate at least once, but total matings per female were not recorded. Male‐paired females in the mid‐life and late‐life treatments reproduced parthenogenetically prior to pairing but sexually once mating occurred (i.e., they switched reproductive modes), whereas females paired with males in early life reproduced sexually throughout their life, as indicated by sex ratios of offspring produced postmating (see Results). Each focal female was paired with a unique virgin male. Males were on average ± *SE* 4.33 ± 0.09 weeks old (from adult molt to pairing). Male age did not differ across male‐paired treatment groups (linear model: F_2,70_ = 1.04, *p* = .36). Where possible, females were paired with males from different breeding stocks to avoid potential inbreeding effects. Females in the female‐paired control group were paired at the same ages as females in the male‐paired treatment but with a 4th‐ or 5th‐instar stock female instead of a male to control for potential density and pairing effects while ensuring only eggs of focal females were sampled. Sample sizes for each treatment combination were as follows: male‐paired: early = 26, mid = 24, late = 23; female‐paired: early = 24, mid = 21, late = 24. The experimental period for focal females (i.e., from the first focal female's adult molt to the last focal female's death) was from January to August 2015. Egg hatching and offspring development (i.e., from the first hatching of eggs to the death of the last offspring) occurred from May 2015 to November 2016.

### 2.3 Effect of female age on mating latency and resistance behaviors

All pairings were initiated at sunset when insects were most active. Following the introduction of male or female partners, we observed behaviors of focal females 10 times per hour over five consecutive hours, with each observation spaced approximately 5 min apart and lasting 15 s, yielding a total of 50 observations per female. Observations were made blind to the age treatment and under red light to limit disturbance. For male‐paired females, we recorded latency to copulate (mins elapsed from pairing onset to copulation) and the frequency of kicking and abdomen shaking/curling. Kicking and shaking/curling were interpreted as resistance behaviors, indicating female reluctance to mate (Arnqvist & Rowe, 2005; Burke et al., 2015). We also tallied frequencies of activity‐ and foraging‐related behaviors which are reported in Supplementary Materials.

We tested for age‐dependent differences in female resistance using generalized linear mixed‐effects models (GLMMs) with binomial error structures and logit link functions. The frequency of kicking and the frequency of abdomen shaking/flicking during mating attempts were the response variables in these analyses, treated as binomial presence–absence proportions. Age at pairing (an ordered, numerical factor) was the fixed effect, with female body length and week of pairing included as scaled covariates, to account for variation in female body size and potential seasonality effects caused by the staggered entry of females into treatment groups, respectively. (In all models, scaling was achieved by subtracting the covariate mean from each covariate value and dividing by the standard deviation). Week of pairing did not confound our analyses since all treatments were represented equally throughout the experimental period, such that the week that focal females entered the experiment at adult eclosion did not differ between treatment combinations (linear model: *F*_3,138_ = 0.628; *p* = .598). Breeder origin of female and breeder origin of partner were included as random effects in these behavioral GLMMs, and an additional observation‐level random effect (OLRE) was also included to correct for overdispersion. Latency to copulate was analyzed using a linear mixed‐effects model (LMM) with a Gaussian error structure and identity link function. Age at pairing was the categorical fixed effect, with scaled covariates for body size and week of pairing, and random effects for female and partner breeder origin, also included.

### 2.4 Effects of sex and switching on female reproductive performance, longevity, and fitness

Over the reproductive lifetime of each focal female, fortnightly subsamples of up to 20 oviposited eggs were placed into hatcheries (see Animal maintenance) and checked three times per week for hatchling emergence. As they emerged, hatchlings were housed in plastic enclosures (12 cm diameter × 21 cm high) with siblings from the same hatchery. Offspring were fed and maintained in these enclosures until they died, at which point they were sexed (if older than 3rd instar) and their developmental age (instar number) recorded. Offspring that reached adulthood were sexed and killed humanely by freezing. Focal females were maintained in their enclosures until they died, whereupon body length was measured with callipers from the tip of the mouth to the end of the ovipositor. We recorded several fitness components for each female, including lifespan, number of eggs, latency to first hatching, egg hatching success, number of eggs developing into adult offspring, number of hatchlings reaching adulthood, offspring sex ratio, total number of adult offspring, and offspring instar at death.

To test for differences in mortality between mated and unmated females of each age class, we analyzed focal female longevity (measured as the number of days from final molt to death) using a LMM with a Gaussian error structure and identity link function. The interacting fixed effects were age at pairing (numerical) and sex of partner (categorical), with breeder origin of female and breeder origin of partner included as random effects, and female body length, week of pairing and lifetime egg output included as scaled covariates. As shorthand, we hereafter use the term “base LMM” to refer to LMMs with this basic model structure. We investigated the hazard rate (i.e., rate of death per day) of focal females by analyzing lifespan using a mixed‐effects Cox model without censoring, using a model structured similarly to female longevity LMM.

To determine the reproductive potential of each reproductive mode, we investigated differences between sex and parthenogenesis by comparing the reproductive performance of females in the early‐life mated treatment that reproduced exclusively sexually with the reproductive performance of females in the early‐life control that reproduced exclusively parthenogenetically. Egg production rate and latency to first hatching (i.e., days from egg collection to first hatching) were analyzed using base LMMs with the egg output covariate excluded. The proportion of eggs hatching, proportion of eggs reaching adulthood, and proportion of hatchlings reaching adulthood were analyzed as presence–absence binomial proportions using GLMMs that were structured similarly to the base LMM except that they were modeled with a binomial error structure and a logit link function, with the egg output covariate excluded, and an additional OLRE included to correct for overdispersion (hereafter, “base GLMM”).

To test for baseload differences in survival between sexually and parthenogenetically produced offspring, the number of offspring from the early‐mated treatment and control dying at first instar was analyzed as a presence–absence binomial proportion in a base GLMM. To assess baseload offspring death rate per day, we analyzed instar number (i.e., developmental age) at death for offspring produced in the early‐mated treatment and control as the uncensored response variable in a mixed‐effects Cox model with sex of partner as the sole fixed factor, with other effects and structure as in the Cox model for focal female hazard.

To test for age‐dependent effects of switching from parthenogenesis to sex, we subtracted prepairing values of reproductive performance from postpairing values and used these differential values for each female as response variables in analyses. Base LMMs with the egg output covariate excluded were used to analyze differential egg production rate, differential mean latency to first hatching, differential proportion of eggs hatched, differential proportion of eggs developing to adulthood, differential proportion of hatchlings developing to adulthood, differential proportion of offspring dying at first instar, and differential rate of adult offspring production.

To test whether offspring hazard differed between treatments that switched to sex at mid‐ or late‐life versus parthenogenetic controls that did not switch, we analyzed offspring instar number at death as an uncensored response variable using a mixed‐effects Cox model with interacting fixed effects of sex of partner (male vs. female), age at pairing (mid‐life vs. late‐life), and offspring origin (prepairing vs. postpairing), and other effects and structure as in the Cox model of focal female hazard.

To determine whether females fertilized their eggs after mating, we used one‐way Student's *t* tests to assess deviations from 0.5 in the sex ratio of offspring produced by male‐paired females following pairing. We also assessed age‐dependent differences in offspring sex ratio using a base GLMM with offspring sex, treated as a binomial male‐to‐female proportion, as the response variable.

To assess the effect of age at mating on overall female fitness, we analyzed lifetime number of adult offspring produced per female across all treatment combinations. Lifetime adult offspring production was treated as a count response variable in a GLMM with Poisson error structures and a square‐root link function, with fixed effects, covariates and mixed effects as in the base GLMM.

To determine the significance of fixed effects and/or their interactions in mixed models, we used likelihood ratio tests (LRTs) that compared each covariate against the full model, and each fixed effect and interaction effect against a reduced model with nonsignificant higher‐order interactions removed. All statistical analyses were performed in R version 3.3.3 (R Core Team 2017). Assumptions for statistical tests were assessed graphically by plotting residuals from models. All LMMs, GLMMs, and Cox models were fitted as random intercept models by maximum likelihood using the lmer or glmer functions in the lme4 package (Bates, Maechler, Bolker, & Walker, 2015), or the coxme function in the coxme package (Therneau, 2015). One‐way *t* tests were performed using the t.test function, and LRTs were performed using the anova function in R. The unit of replication for all analyses was the focal female.

## 3 RESULTS

### 3.1 Mating latency and resistance behaviors

**Figure 2:**
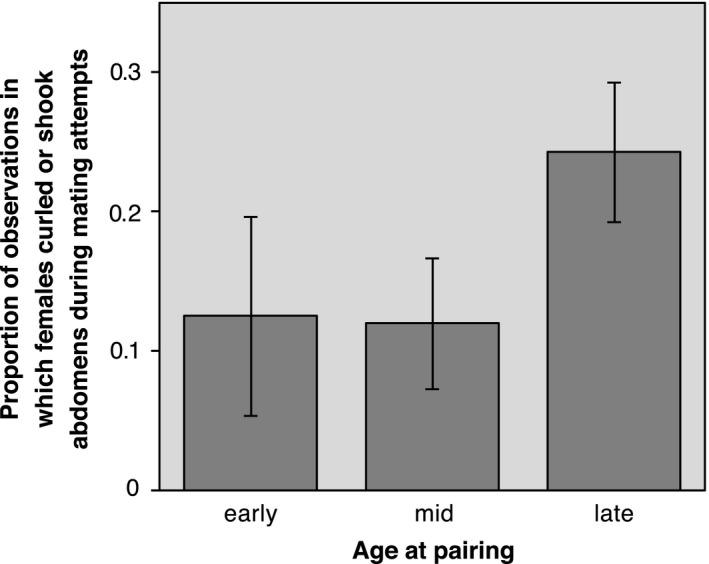
Bar graph showing the proportion of observations (means ± *SE*s) in which females in the male‐paired treatment curled or shook their abdomen during mating attempts

There was no effect of female age on latency to copulate, indicating that males found females of different ages equally attractive and/or females were equally receptive at each age. Body size and seasonality (week of pairing) also had no influence on copulation latency (Table 1). However, the frequency of kicking during mating attempts increased with increasing female age, even though, overall, kicking decreased over the experimental period (Table 1). Likewise, abdomen curling/shaking during mating attempts was more frequent in older than younger females (Figure 2), but body size and week of pairing had no effect on this behavior (Table 1). Together, these results suggest that older females were more resistant to mating than younger females.

**Table 1:** Statistical output from LMM, GLMM, and Cox model analyses of resistance‐related behaviors, female longevity and hazard, and offspring sex ratio

Model effect	Response variable											
	Latency to copulate		Kicking at males		Curling/shaking abdomen		Female longevity		Female hazard		Postpairing offspring sex ratio	
	LMM	LRT	GLMM	LRT	GLMM	LRT	LMM	LRT	Cox model	LRT	GLMM	LRT
Sex of partner (male)	NA	NA	NA	NA	NA	NA	−4.91 (5.54)	0.76 **0.38**	−0.13 (0.49) *0.88*	0.02 **0.88**	NA	NA
Age at pairing	−0.15 (0.26)	0.45 **0.50**	1.20 (0.48)	7.12 **0.01**	1.28 (0.53)	6.28 **0.01**	−1.92 (2.02)	0.29 **0.59**	0.11 (0.16) *1.12*	1.01 **0.32**	0.01 (0.23)	0.002 **0.97**
Sex (male) × Age interaction	NA	NA	NA	NA	NA	NA	2.20 (2.58)	0.07 **0.79**	0.05 (0.22) *1.05*	0.05 **0.83**	NA	NA
Body length	−0.04 (0.19)	0.03 **0.87**	−0.25 (0.33)	0 **1.00**	−0.08 (0.39)	0.05 **0.83**	−0.75 (1.11)	0.48 **0.49**	0.05 (0.08) *1.06*	0.41 **0.52**	−0.35 (0.18)	4.00 **0.05**
Week of pairing	0.33 (0.25)	2.08 **0.15**	−1.44 (0.50)	10.27 **0.001**	0.18 (0.48)	0.15 **0.70**	6.82 (1.28)	19.23 **<0.001**	−0.51 (0.13) *0.60*	11.51 **<0.001**	0.09 (0.19)	0.24 **0.63**
Lifetime egg output	NA	NA	NA	NA	NA	NA	10.54 (1.14)	69.48 **<0.001**	−*0.76 (0.10)* *0.47*	55.96 **<0.001**	NA	NA

Values given for LMMs and GLMMs are model coefficients and standard errors (in brackets).

Hazard ratios for the Cox model are in italics.

Values given for LRTs are chi‐square statistics (df = 1) and *p* values (in bold).

NAs indicate model effects not included in analyses.

Shaded cells rows indicate significant effects according to LRTs.

Results for foraging and activity‐related behaviors, plus summary statistics for all behavioral responses, are provided in Supplementary Material.

### 3.2 Female longevity and survival

**Figure 3:**
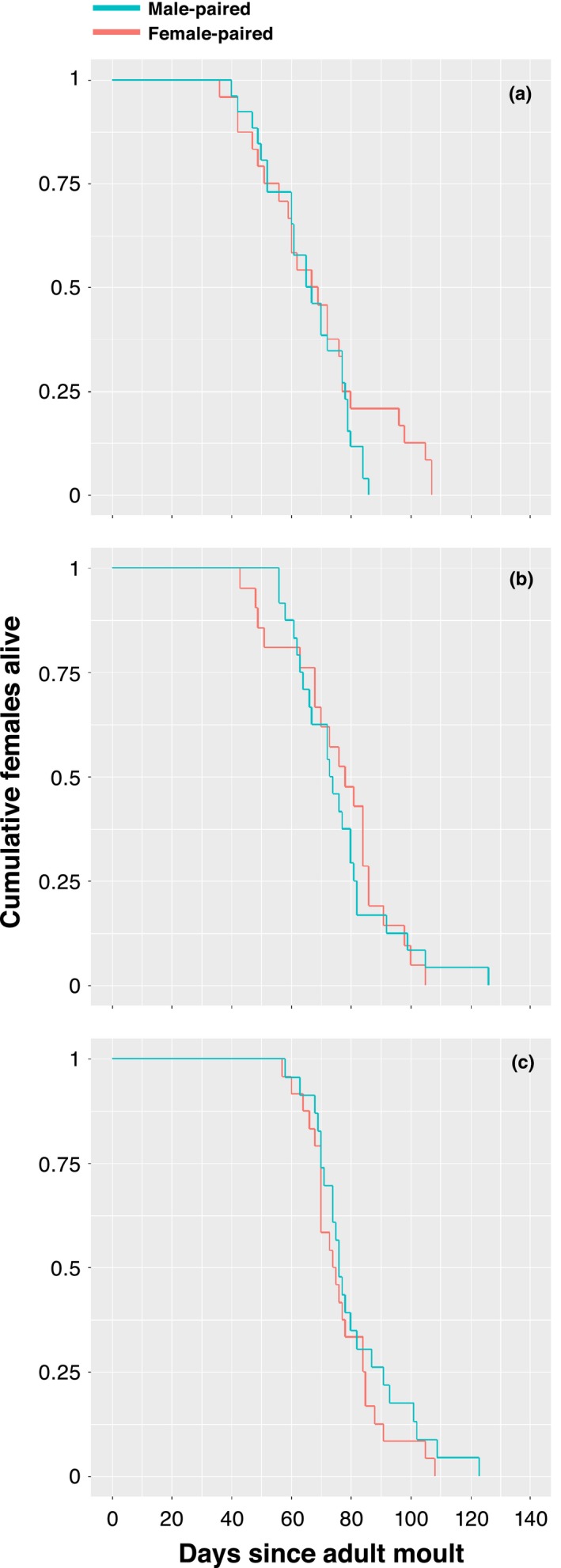
Female survival curves showing the proportion of females alive in the early‐life treatment and control (a), the mid‐life treatment and control (b), and the late‐life treatment and control (c)

Female longevity was unaffected by sex of partner, age at pairing, or their interaction (Table 1). Female body size did not affect longevity, but females lived longer when paired later in the experimental period (Table 1). Longer‐lived females also produced more eggs than shorter‐lived individuals (Table 1). Survival analysis showed that the probability of death per day (hazard) was also nonsignificantly affected by age at pairing, sex of partner, or their interaction (Figure 3; Table 1). Body size did not covary with female hazard, but week of pairing and lifetime egg output were both significantly negatively correlated with hazard (Table 1). Across all females, we found a strong positive correlation between longevity and egg output (*r *=* *0.56, *p *<* *.001). Thus, there is no obvious trade‐off between lifespan and fecundity in this system.

### 3.3 Reproductive performance of sexual versus parthenogenetic females

**Figure 4:**
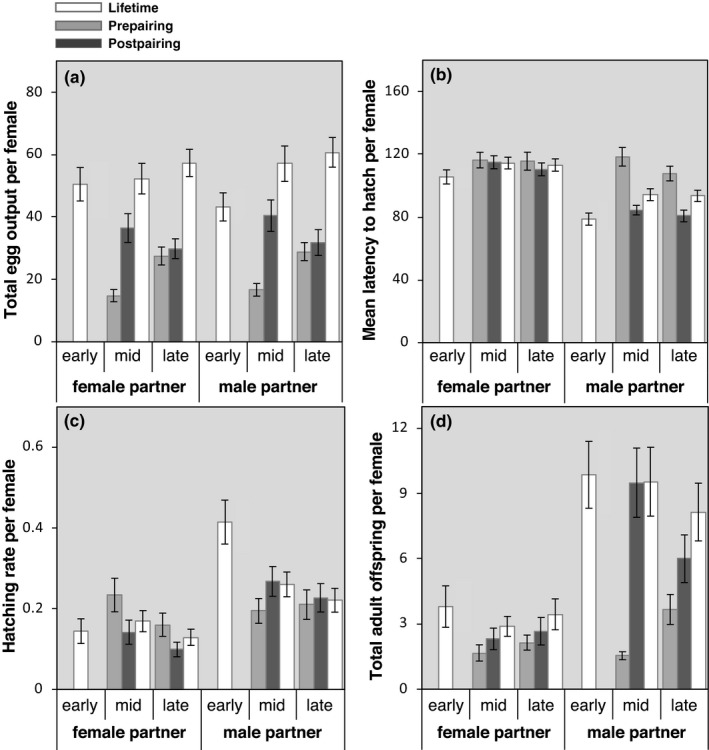
Per‐female means ± *SE*s for total egg output (a), mean latency to first hatching (b), hatching rate (c), and total production of adult offspring (d) for each treatment combination pre‐ and postpairing. Lifetime means and *SE*s are also shown (in white). Females in the early pairing group produced no eggs prepairing since pairings in this treatment occurred prior to the onset of oviposition, so only lifetime means are shown for these females (see Materials and Methods)

**Figure 5:**
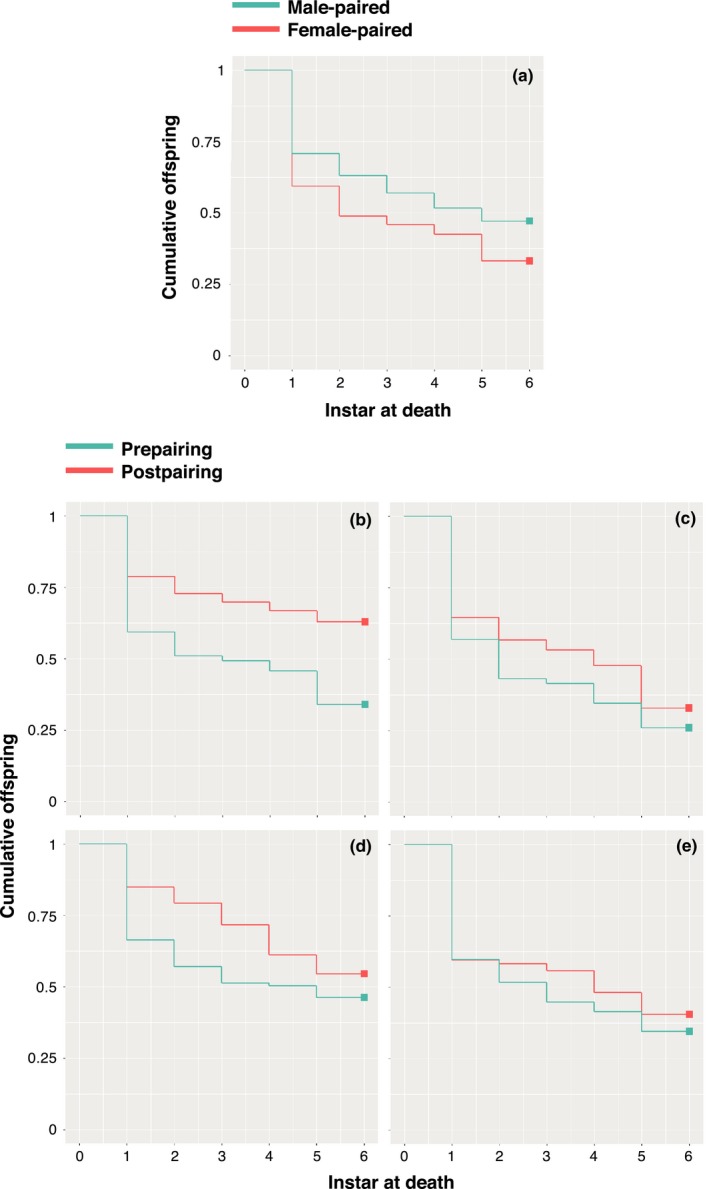
Offspring survival curves showing the proportion of offspring alive in the early‐life treatment and control (a), the mid‐life, male‐paired treatment (b), the mid‐life, female‐paired control (c), the late‐life, male‐paired treatment (d), and the late‐life, female‐paired control (e). Offspring that reached adulthood are indicated by instar 6 on each of the *x*‐axes and by bold squares on the survival curves

Eggs of females that reproduced sexually throughout their lives had shorter mean latencies to first hatching (Figure 4b) and hatched at a higher rate (Figure 4c) than eggs produced by females that reproduced parthenogenetically throughout their lives (Table 2). Sexually produced eggs and hatchlings were also more likely to develop and survive to adulthood (Table 2). However, egg production rate did not differ between sexual and parthenogenetic females (Figure 4a; Table 2). Maternal week of pairing and body size did not covary with any of these performance‐related response variables, but larger females produced offspring with lower egg‐to‐adult viability (Table 2). A larger proportion of offspring died as first‐instar nymphs when produced parthenogenetically than when produced sexually (Figure S1; Table 2). However, hazard rates did not differ between sexually and parthenogenetically produced offspring (Figure 5a; Table 2). Body size and maternal week of pairing had no effect on the proportion of offspring dying at first instar or the hazard rate of offspring (Table 2). Overall, these results suggest that sexual reproduction had higher reproductive potential than parthenogenesis.

**Table 2:** Statistical output from LMM, GLMM, and Cox model analyses of reproductive performance and offspring mortality of females that reproduced sexually versus parthenogenetically throughout life

Model effect	Response variable															
	Daily egg production rate		Mean latency to first hatching		Proportion of eggs hatched		Proportion of eggs reaching adult instar		Proportion of hatchlings reaching adult instar		Proportion of offspring dying at first instar		Hazard of offspring produced sexually versus asexually		Lifetime adult offspring production	
	LMM	LRT	LMM	LRT	GLMM	LRT	GLMM	LRT	GLMM	LRT	GLMM	LRT	Cox model	LRT	GLMM	LRT
Sex of partner (male)	−0.12 (0.22)	0.31 **0.58**	−28.74 (5.78)	17.33 **<0.001**	1.99 (0.41)	20.04 **<0.001**	1.75 (0.36)	18.54 **<0.001**	0.79 (0.36)	4.62 **0.03**	−0.81 (0.26)	9.79 **0.002**	−0.32 (0.16) *0.73*	2.61 **0.11**	1.50 (0.51)	32.25 **<0.001**
Age at pairing	NA	NA	NA	NA	NA	NA	NA	NA	NA	NA	NA	NA	NA	NA	0.16 (0.18)	0.37 **0.54**
Sex (male) × Age interaction	NA	NA	NA	NA	NA	NA	NA	NA	NA	NA	NA	NA	NA	NA	−0.15 (0.24)	0.43 **0.51**
Body length	−0.02 (0.11)	0.03 **0.85**	1.83 (2.87)	0.40 **0.53**	−0.37 (0.20)	3.46 **0.06**	−0.40 (0.18)	4.85 **0.03**	−0.16 (0.17)	0.86 **0.35**	−0.09 (0.11)	0.72 **0.40**	0.07 (0.07) *1.07*	0.81 **0.37**	−0.02 (0.10)	0.05 **0.82**
Week of pairing	0.06 (0.11)	0.31 **0.58**	4.21 (2.88)	1.32 **0.25**	−0.19 (0.22)	0.75 **0.39**	−0.26 (0.18)	2.04 **0.15**	−0.004 (0.18)	<0.001 **0.98**	0.25 (0.18)	2.06 **0.15**	0.04 (0.09) *1.04*	0.11 **0.74**	−0.16 (0.14)	1.44 **0.23**

Values given for LMMs and GLMMs are model coefficients and standard errors (in brackets).

Hazard ratios for the Cox model are in italics.

Values given for LRTs are chi‐square statistics (df = 1) and *p* values (in bold).

NAs indicate model effects not included in analyses.

Shaded cells indicate significant effects according to LRTs.

### 3.4 Reproductive performance of switching females versus nonswitching controls

Switching to sex after a period of parthenogenetic reproduction shortened latency to first hatching and increased hatching success (Figure 4c; Table 3). Switching also increased the proportion of eggs reaching adulthood compared to parthenogenetic controls, regardless of age at switching (Table 3). These patterns suggest that mating rather than pairing was responsible for higher performance of females that switched. The absence of age‐at‐pairing effects in these analyses suggests that females gained benefits of switching regardless of whether they switched at mid‐ or late‐life. Switching had no effect on egg production rate or the proportion of hatchlings reaching adulthood (Table 3). However, females paired in late life had a higher differential egg production rate than females paired in mid‐life irrespective of the sex of the partner (Figure 4a; Table 3), indicating that old‐age exposure to a conspecific of any sex had a beneficial effect on reproductive rate. For all other measures of reproductive performance mentioned above, age at pairing and the interaction between age at pairing and sex of partner had no effect (Table 3). The only significant covariate in analyses of these performance measures was maternal week of pairing: The differential proportion of eggs hatching successfully decreased over the pairing period of the experiment (Table 3).

**Table 3:** Statistical output from LMM, GLMM, and Cox model analyses of differential reproductive performance and offspring mortality of females that switched versus did not switch to sex

Model effect	Response variable															
	Differential daily egg production rate		Differential mean latency to first hatching		Differential proportion of eggs hatching		Differential proportion of eggs reaching adult instar		Differential proportion of hatchlings reaching adult instar		Differential proportion of offspring dying at first instar		Hazard of offspring produced by switching versus control females		Differential lifetime adult offspring production	
	LMM	LRT	LMM	LRT	LMM	LRT	LMM	LRT	LMM	LRT	LMM	LRT	Cox model	LRT	GLMM	LRT
Sex of partner (male)	0.33 (1.09)	0.59 **0.44**	−46.02 (31.08)	15.45 **<0.001**	0.43 (0.22)	14.23 **<0.001**	0.33 (0.15)	8.14 **0.004**	0.15 (0.53)	0.15 **0.70**	0.50 (0.53)	1.17 **0.28**	−0.92 (0.44) *0.40*	15.14 **<0.001**	2.26 (1.40)	10.34 **<0.001**
Age at pairing	0.67 (0.31)	6.86 **0.009**	−4.60 (8.75)	0.01 **0.93**	0.04 (0.06)	0.07 **0.79**	0.01 (0.04)	1.17 **0.28**	−0.05 (0.15)	0.72 **0.40**	0.16 (0.15)	0.76 **0.38**	−0.13 (0.15) *0.88*	0.26 **0.61**	−0.98 (0.42)	32.29 **<0.001**
Offspring origin (prepairing)	NA	NA	NA	NA	NA	NA	NA	NA	NA	NA	NA	NA	0.22 (0.59) *1.25*	11.41 **<0.001**	NA	NA
Sex (male) × Age interaction	−0.20 (0.43)	0.21 **0.64**	7.85 (12.13)	0.42 **0.52**	−0.10 (0.09)	1.45 **0.23**	−0.09 (0.06)	2.47 **0.12**	−0.08 (0.21)	0.14 **0.71**	−0.15 (0.21)	0.59 **0.44**	0.23 (0.18) *1.26*	0.84 **0.36**	−0.57 (0.52)	1.15 **0.28**
Sex (male) × Origin (pre) interaction	NA	NA	NA	NA	NA	NA	NA	NA	NA	NA	NA	NA	0.84 (0.76) *2.31*	0.66 **0.42**	NA	NA
Origin (pre) × Age interaction	NA	NA	NA	NA	NA	NA	NA	NA	NA	NA	NA	NA	−0.02 (0.23) *0.98*	1.54 **0.21**	NA	NA
Sex (male) × Age × Origin (pre) interaction	NA	NA	NA	NA	NA	NA	NA	NA	NA	NA	NA	NA	−0.28 (0.29) *0.46*	0.90 **0.35**	NA	NA
Body length	−0.14 (0.11)	1.44 **0.23**	0.46 (3.12)	0.02 **0.88**	−0.02 (0.02)	0.82 **0.36**	0.01 (0.02)	0.19 **0.66**	0.03 (0.06)	0.21 **0.65**	−0.02 (0.05)	0.23 **0.63**	0.001 (0.03) *1.00*	0.003 **0.96**	−0.04 (0.13)	0.09 **0.76**
Week of pairing	0.11 (0.11)	0.97 **0.32**	1.25 (3.09)	0.16 **0.69**	−0.05 (0.02)	4.23 **0.04**	−0.02 (0.02)	1.57 **0.21**	−0.10 (0.06)	3.47 **0.06**	0.11 (0.05)	4.14 **0.04**	−0.08 (0.04) *0.92*	5.59 **0.02**	−0.45 (0.16)	8.56 **0.003**

Values given for LMMs and GLMMs are model coefficients and standard errors (in brackets).

Hazard ratios for the Cox model are in italics.

Values given for LRTs are chi‐square statistics (df = 1) and *p* values (in bold).

NAs indicate model effects not included in analyses.

Shaded cells indicate significant effects according to LRTs.

Switching had no effect on the proportion of offspring dying at first instar (Table 3). Age at pairing, the interaction between sex of partner and age at pairing, and body size also had no effect on the differential proportion of offspring dying at first instar (Table 3). However, the differential proportion of offspring dying at first instar increased as maternal week at pairing increased (Table 3). Holding all other variables constant, maternal week at pairing also influenced the hazard of offspring produced by mid‐ and late‐paired females, with probability of death from hatching to final molt decreasing by 8% with each increase in maternal week of pairing (Table 3). Switching to sex resulted in an overall reduction of 60% in offspring hazard relative to offspring of control females (Figure 5b–e; Table 3). The age at which females were paired had no effect on offspring hazard (Table 3). However, offspring that hatched from eggs produced postpairing exhibited hazard rates 25% lower than offspring hatched from eggs produced prepairing (Table 3), and this effect occurred regardless of whether pairing was with a male or a female (i.e., there was no offspring origin × sex of partner interaction effect; Table 3). This suggests that exposure to conspecifics of either sex during oviposition in mid‐ or late‐life significantly enhanced offspring survival.

### 3.5 Offspring sex ratio

As expected, all offspring from unmated controls were female. The sex ratios of offspring produced after mating were not significantly different from 0.5 (one‐way *t* test: early‐life: *t*₂₁ = 1.005, *p* = .327; mid‐life: *t*₁₉ = 1.414, *p* = .174; late‐life: *t*_17_ = 1.390, *p* = .183; Table S2), suggesting that most offspring from pairings with males were sexually produced. Age at pairing did not affect the sex ratio of offspring produced after mating, but larger females produced a significantly higher ratio of male offspring (Table 1).

### 3.6 Adult offspring

Females that reproduced sexually at any point in their lives produced significantly more adult offspring than females that reproduced only via parthenogenesis (Figure 4d; Table 2). Age at pairing had no effect on the number of adult offspring that females produced (Table 2). Surprisingly, however, females that mated earliest, and therefore had the greatest opportunity to reproduce sexually, did not produce more offspring over their lifetime than females that mated later in life and that therefore had less time to reproduce sexually (i.e., there was no interaction between age at pairing and sex of partner; Table 2).

Females that switched to sex produced more adult offspring from eggs laid postpairing than from eggs laid prepairing, and this differential was significantly higher than that of parthenogenetic controls that did not switch (Figure 4d; Table 3). Females paired in mid‐life produced more adult offspring from eggs laid postpairing versus prepairing than females paired in late life (Figure 4d; Table 3). However, there was no interaction between age at pairing and sex of partner (Table 3), suggesting that females that switched to sex later in life did not upregulate oviposition rate or offspring viability to a greater extent than females paired at mid‐life. Rather, early‐mated females laid fewer eggs than females that switched to sex in mid‐ and late‐life (see Figure 4a). The differential number of offspring produced before versus after pairing by females paired in mid‐ and late‐life was also significantly correlated with maternal week at pairing, with fewer adult offspring produced by females the later in the season that pairings took place.

Summary statistics for all fitness components are provided in Tables S1 and S2 in Supplementary Material.

## 4 DISCUSSION

We found little evidence that either sexual reproduction alone or switching to sex after a period of parthenogenetic reproduction was costly to *S. larryi* females. Sexual reproduction consistently boosted adult offspring counts relative to parthenogenetic controls regardless of the level of females’ prior investment in parthenogenetic reproduction. We found no evidence of a reproduction‐longevity trade‐off: Sex and switching enhanced fitness without affecting female mortality. These results suggest that *S. larryi* does not experience strong sexual conflict over reproductive mode or timing of mating. Although we identified potential disadvantages of mating early and late that were symptomatic of sexual conflict, our results overall suggest that the benefits of sexual reproduction and reproductive switching in *S. larryi* outweigh any potential costs. We also observed a positive effect of female–female pairing on egg output and offspring viability, indicating a socially mediated maternal effect.

Females reproducing parthenogenetically throughout life incurred significant fitness costs including lower egg hatching rate, higher offspring hazard rate throughout development, lower egg‐to‐adult viability, lower hatchling‐to‐adult viability, and lower adult offspring counts. Females gained significant benefits by switching from parthenogenesis to sex regardless of the age at switching. Switching shortened mean latency to first hatching, improved hatching rates, and increased egg‐to‐adult viability and the rate of adult offspring production. These results suggest that sex at any age is beneficial. However, switching later provided an equivalent fitness benefit to switching earlier. This was surprising given that females that mated in late life had a shorter window of opportunity to boost fitness via sex. Although our late‐life treatment may not have been late enough to detect costs of switching at an older age, this is an unlikely explanation because most females in the late male‐paired treatment spent more than half their reproductive lives reproducing parthenogenetically prior to switching. Rather, our results suggest that switching to sex in later life may be as beneficial to fitness as sexual reproduction throughout life because of costs associated with early mating. Females that mated early produced the fewest eggs of any treatment combination, which suggests that mating prior to oviposition may reduce the number of eggs laid. It is possible that these apparent costs were due to recently eclosed females not being fully mature when paired with males (e.g., see Maklakov, Kremer, & Arnqvist, 2007). Yet, early‐mated females were the least resistant to mating, and the enhanced performance of sexually produced offspring compensated for the reduction in fecundity in these females.

The increased frequency at which older females resisted males by kicking and by curling or shaking their abdomens suggests that switching to sex in late life may be disadvantageous in *S. larryi*. We did not observe females escaping from males by walking or flying away, but it is possible that our experimental conditions affected females’ ability to do so. However, we found little evidence of fitness costs of switching in late life. Males did not cause older females to be less active or less willing to forage, and there was no effect of female age on copulation latency. This suggests that sexual conflict over some other trait, such as mating duration, could be driving female resistance. Alternatively, females might easily escape unwanted mating attempts in natural settings, but small laboratory cages might have prevented females from resisting effectively. Although we expected resistance to correlate with costs, mating (switching) did not reduce egg output or increase mortality in older females. However, switching failed to enhance hatchling‐to‐adult viability, which suggests that some of the positive effects of mating on offspring performance that we observed when females mated in early life may not be obtainable when females mate at later ages. A previous study on the phasmatid *E. tiaratum* investigated outcomes of reproductive switching at a single age and found significant mortality and fecundity costs which were also associated with striking resistance behaviors, including kicking, abdomen curling, and male‐repellent secretions (Burke et al., 2015). The contrasting absence of significant mating costs in *S. larryi* suggests that sexual conflict may be less important in *S. larryi* than in *E. tiaratum*.

Sexual conflict over reproductive mode could potentially manifest at the gametic level in facultative systems if females produce eggs resistant to fertilization (Burke & Bonduriansky, 2017a). This could result in a partial or incomplete switch to sex whereby some eggs develop parthenogenetically after females mate. Low rates of this kind of postcopulatory parthenogenesis have been reported in a facultatively asexual fly (Chang et al., 2014), stick insect (Arbuthnott et al., 2015), and termite (Yashiro & Matsuura, 2014). If *S. larryi* has a similar capacity to reproduce parthenogenetically after mating, we would expect offspring of mated females to exhibit female‐biased sex ratios. Although offspring sex ratios from mated treatments did not deviate significantly from 0.5, estimates were female‐biased in each case (see Table S2), suggesting that some eggs may have remained unfertilized after mating or that offspring mortality was male‐biased. Offspring sex ratios also became more female‐biased (although not significantly so) as female age at mating increased (see Table S2), which suggests that females that mate later in life may be more likely to produce unfertilized eggs. This could occur if older females’ eggs are more resistant to fertilization, or if spermathecae are less efficient at retaining sperm. Whether incomplete switching in facultatively asexual organisms is mostly driven by sperm limitation or sexual conflict remains an open question. Molecular assessment of offspring parentage and examination of female sperm stores could help to provide answers.

Interestingly, although mating consistently boosted reproductive performance in *S. larryi*, the survival of offspring produced by mid‐ and late‐paired females was also significantly enhanced by pairing with another female. This suggests a maternal effect on offspring survival, mediated by social stimulation by conspecifics. *S. larryi* females may benefit by reproducing parthenogenetically some of the time, especially if population density is low or males are rare (see Schwander, Vuilleumier, Dubman, & Crespi, 2010). However, females may need to be physiologically stimulated by male hormones, pheromones or physical contact to reproduce maximally, which is a common characteristic of many internally fertilized sexual species, especially mammals (Neiman, 2004). For facultatively asexual females that require male stimulation for maximal reproduction, recurrent isolation from males could significantly constrain fitness. But females could potentially overcome this constraint by sourcing equivalent stimulation from conspecific females when males are absent. This apparently occurs in the gynogenetic whiptail, *Cnemidophorus uniparens*, where female‐to‐female pseudocopulation stimulates parthenogenetic egg development in the absence of male stimulation (Crews & Young, 1991). Although pseudocopulation does not occur in *S. larryi*, the beneficial effect of conspecific exposure on survival of parthenogenetically produced offspring that we observed in this species is likely mediated by factors common to both sexes, such as allomones, cuticular hydrocarbons, or physical contact.

Our finding that a solely parthenogenetic strategy conferred lower fitness than a solely sexual one is consistent with previous studies on other facultative systems. In many facultatively parthenogenetic insects, parthenogenesis results in lower reproductive performance compared to sex, manifesting as depressed fecundity (Chang et al., 2014), poor offspring viability (Corley & Moore, 1999), and/or reduced offspring lifespan (Kramer & Templeton, 2001). *S. larryi* appears to be no different in this regard, with parthenogenetically produced offspring performing less successfully than sexually produced offspring. This widespread pattern of low parthenogenetic success in facultatively asexual insects (see Lamb & Willey, 1979) has led to suggestions that developmental, genetic, ecological or evolutionary constraints could prevent parthenogenesis evolving from sexual ancestors (Burke & Bonduriansky, 2017a; Corley et al., 1999; Engelstadter, 2008; Lehtonen, Kokko, & Parker, 2016; Neiman, 2004; Vrijenhoek, 1989). For example, several preadaptations at the cellular level have been identified as essential for optimal parthenogenetic development (Engelstadter, 2008), and mechanisms that maintain genetic heterozygosity between generations are thought to be crucial for long‐term success of parthenogens (Simon, Delmotte, Rispe, & Crease, 2003). Meiosis has also been suggested as an important constraint on the evolution of parthenogenesis in multicellular Eukaryotes (Levitis, Zimmerman, & Pringle, 2017). However, the specific factors that limit performance of parthenogenetically produced offspring in *S. larryi* remain unknown.

Nevertheless, the fact that the capacity for parthenogenesis is universal in S. *larryi*—in contrast to tychoparthenogenetic species where unfertilized eggs spontaneously develop only rarely and by accident (Markow, 2013; Normark & Kirkendall, 2009)—suggests that parthenogenetic reproduction is selectively favorable in at least some circumstances. The value of facultative parthenogenesis may lie in the capacity of females to attain some fitness when mates are unavailable (Gerritsen, 1980; Schwander et al., 2010). The fact that switching from parthenogenesis to sex in *S. larryi* was beneficial at any age and that young females rarely exhibited resistance behaviors, supports this idea. Thus, the capacity for reproductive switching might persist in *S. larryi* due to recurrent mate limitation rather than sexual conflict. Field studies of natural populations will be required to test this hypothesis.

## 5 CONCLUSION

Our study is the first to investigate the effects of age at switching on female fitness in a facultatively parthenogenetic system. We found that *S. larryi* females were receptive to mating in early life despite producing fewer eggs after mating. Female resistance increased with age, even though switching at older ages did not negatively affect female survival or offspring performance. Overall, sexually produced offspring outperformed parthenogens, regardless of the age at which females switched to sex, supporting the theoretical prediction that some sex is better than none (D'Souza & Michiels, 2010). Interestingly, however, we also found that exposure to conspecific females at any age enhanced reproductive performance and the survival of parthenogenetic offspring, suggesting that females may use reproductive stimulation from other females to limit reliance on male stimuli. Taken together, our findings show that age at switching can have subtle effects on fecundity and offspring performance that could have important context‐dependent effects in natural environments. Given the pronounced mating costs previously observed in the stick insect *E. tiaratum* (see Burke et al., 2015) and the apparent lack of costs reported here for *S. larryi,* our study also suggests that sexual conflict over reproductive switching can vary markedly among facultative species.

## AUTHOR CONTRIBUTIONS AND ACKNOWLEDGMENTS

The authors thank A. Hooper, E. Macartney, Z. Wylde, and three anonymous reviewers for helpful comments on earlier drafts of the manuscript. NWB and RB jointly contributed to the conception and design of the study, the interpretation of the data, and the drafting and revising of the manuscript. NWB collected and analyzed data. Both authors approved the final version of the manuscript at publication and take responsibility for the work's integrity and accuracy.

## CONFLICT OF INTEREST

None declared.
