# Supplementary material for: The fitness effects of delayed switching to sex in a facultatively asexual insect
Source: Ecol Evol. 2018 Feb 6;8(5):2698–711. doi: 10.1002/ece3.3895 (PMC5838058; doi:10.1002/ece3.3895)
Supplement: Supplementary file 1 [file ECE3-8-2698-s001.docx]

# APPENDIX

## Activity and foraging behaviours

We tested for age-dependent male effects on female activity- and foraging-related behaviours during pairings (but prior to onset of copulation) using generalised linear mixed effects models (GLMMs) with binomial error structures and logit link functions. Frequencies of inactivity, locomotion, ovipositor probing and leaf-eating were the response variables in these analyses, each treated as binomial presence-absence proportions. Age at pairing (numerical) and sex of partner (categorical) were the interacting fixed effects, with female body length and week of pairing included as scaled covariates. Breeder origin of female and breeder origin of partner were the random effects, with an additional observation-level random effect (OLRE) included to correct for overdispersion.

All measures of female activity and foraging behaviour obtained prior to mating were significantly affected by female age at pairing. Females paired at older ages, regardless of the sex of their partner, were less inactive, more locomotive, more likely to be observed eating leaves and probing their ovipositor in search of oviposition sites than females paired at younger ages (Table S3). Male-paired females were more inactive, less locomotive, and less likely to forage prior to mating than female-paired controls, but sex of partner had no effect on the frequency of ovipositor probing (Table S3). The interaction between age at pairing and sex of partner had no significant effect on inactivity, locomotion, probing or foraging; nor was there an effect of female body size on these behaviours (Table S3). Week of pairing affected some behaviours, with inactivity increasing and foraging decreasing over the experimental period (Table S3). This effect was most likely due to seasonal declines in night-time temperatures experienced by focal females over the experimental period from January to August (Summer to Winter in the southern hemisphere), causing reduced activity levels and foraging rates.

## Figure S1

Juvenile offspring developmental stage (instar) at death. Offspring counts are pooled by treatment combination rather than female to calculate proportions for this figure. Females in the early pairing group produced no eggs prior to pairing because pairings in this treatment occurred before the onset of oviposition (see Material and Methods).


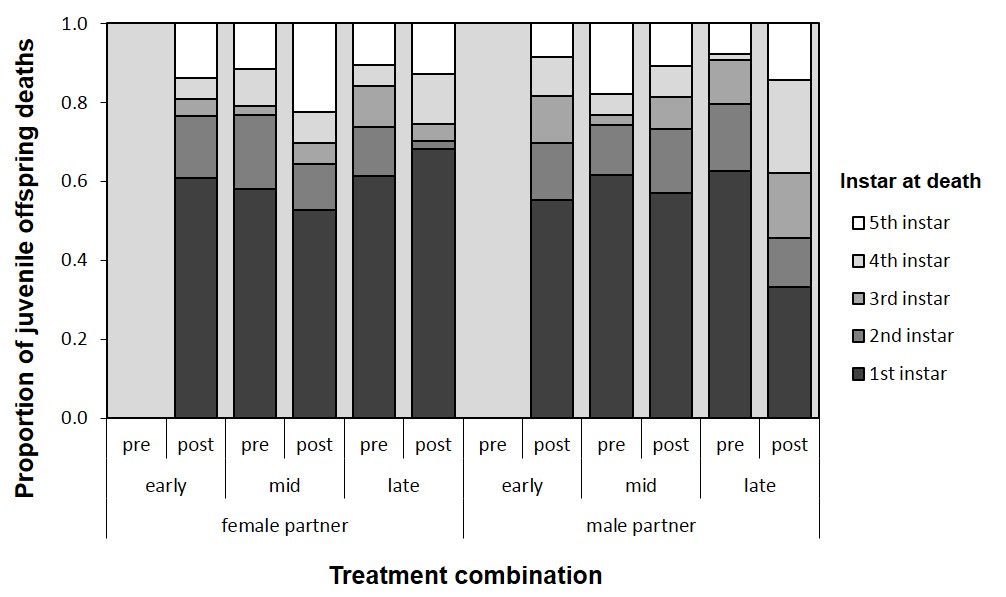


## Table S1

Summary statistics for female behaviours.

|  | **Early-life pairing** | | **Mid-life pairing** | | **Late-life pairing** | |
| --- | --- | --- | --- | --- | --- | --- |
|  | **With male** | **With female** | **With male** | **With female** | **With male** | **With female** |
| **Inactivity** | 30.1 (16.0; 25) | 40.5 (9.74; 22) | 27.9 (15.3; 23) | 40.4 (12.3; 21) | 24.8 (16.0; 23) | 31.3 (13.6; 24) |
| **Locomotion** | 1.08 (3.34; 25) | 3.59 (6.82; 22) | 3.83 (6.81; 23) | 4.76 (7.23; 21) | 1.65 (3.14; 23) | 8.38 (8.23; 24) |
| **Probing ovipositor** | 0.00 (0.00; 25) | 0.00 (0.00; 22) | 1.52 (5.04; 23) | 1.62 (5.24; 21) | 0.65 (2.01; 23) | 4.00 (6.59; 24) |
| **Eating leaves** | 0.56 (1.39; 25) | 2.27 (4.85; 22) | 1.26 (4.18; 23) | 1.90 (3.91; 21) | 0.65 (1.27; 23) | 5.25 (6.13; 24) |
| **Kicking at males** | 3.06 (4.57; 16) | NA | 1.86 (2.86; 14) | NA | 3.59 (3.91; 17) | NA |
| **Curling/shaking abdomen** | 2.19 (5.08; 16) | NA | 3.86 (6.11; 14) | NA | 6.65 (5.82; 17) | NA |
| **Latency to copulate (hrs)** | 1.78 (0.98; 15) | NA | 2.14 (1.37; 13) | NA | 1.89 (1.13; 17) | NA |

Values given are mean (standard deviation; sample size).

Means for activity- and foraging-related behaviours represent the average number of observations that females were inactive, locomotive, probed their ovipositors and ate leaves during pairings (i.e., prior to any copulation).

Means for resistance-related behaviours represent the average number of observations that male-paired females kicked their legs and curled or shook their abdomens during mating attempts.

No mating-related behaviours were observed in female-paired treatments, as indicated by NA.

Mean latency to copulate represents the average time taken for male-paired females to mate within the 5-hour behavioural observation period; females that mated outside this period are not included in these copulation latency statistics.

## Table S2

Summary statistics for measures of reproductive performance.

|  | **Early-life pairing** | | **Mid-life pairing** | | **Late-life pairing** | |
| --- | --- | --- | --- | --- | --- | --- |
|  | **With male** | **With female** | **With male** | **With female** | **With male** | **With female** |
| **Female lifespan (days)** | 65.7 (13.7; 26) | 69.5 (21.2; 24) | 76.0 (16.6; 24) | 75.5 (17.5; 21) | 81.2 (15.6; 23) | 77.0 (12.6; 24) |
| **Lifetime egg output** | 43.2 (23.0; 26) | 50.5 (26.2; 24 | 57.1 (27.8; 24) | 52.3 (22.5; 21) | 60.7 (22.8; 23) | 57.3 (21.3; 24) |
| **Post-pairing offspring sex ratio (prop. ♀)** | 0.56 (0.26; 22) | 1.00 (0.00; 16) | 0.57 (0.22; 20) | 1.00 (0.00; 18) | 0.59 (0.27; 18) | 1.00 (0.00; 15) |
| **Adult offspring count** | 8.35 (7.54; 26) | 2.38 (3.44; 24) | 8.75 (7.59; 24) | 2.48 (2.06; 21) | 7.09 (6.19; 23) | 2.58 (3.01; 24) |
| **Egg production rate (per day)** | 1.07 (0.51; 26) | 1.11 (0.34; 24) | -0.08 (0.86; 24) | -0.05 (0.74; 21) | 0.03 (0.73; 22) | 0.22 (0.87; 24) |
| **Mean latency to first hatching (days)** | 78.7 (18.8; 24) | 105.6 (18.5; 17) | -32.4 (27.6; 19) | -2.19 (25.2; 15) | -29.2 (21.2; 17) | -6.74 (28.1; 18) |
| **Prop. of eggs hatching** | 0.41 (0.28; 26) | 0.14 (0.15; 24) | 0.15 (0.21; 24) | -0.07 (0.21; 21) | 0.09 (0.27; 22) | -0.03 (0.16; 24) |
| **Prop. of eggs reaching adult instar** | 0.22 (0.17; 24) | 0.05 (0.05; 17) | 0.14 (0.18; 24) | -0.02 (0.15; 21) | 0.06 (0.16; 22) | -0.005 (0.06; 24) |
| **Prop. of hatchlings reaching adult instar** | 0.44 (0.25; 24) | 0.32 (0.25; 17) | 0.14 (0.56; 19) | 0.09 (0.53; 16) | 0.01 (0.36; 18) | 0.05 (0.42; 18) |
| **Differential adult offspring count** | NA | NA | 7.08 (7.37; 24) | 1.05 (2.48; 21) | 2.30 (4.83; 23) | 0.08 (1.86; 24) |

Values given are mean (standard deviation; sample size).

Statistics highlighted in grey are for differential measures of reproductive performance (i.e., the difference between post- and pre-pairing performance).

Negative mean differential values indicate that, on average, post-pairing values per female were lower than pre-pairing values.

NAs indicate treatments for which certain statistics do not apply.

## Table S3

Statistical output for GLMM analyses of behaviours during pairings (i.e., prior to any copulations).

| **Response variable**  **Model**  **effect** | **Inactivity** | | **Locomotion** | | **Probing ovipositor** | | **Eating leaves** | |
| --- | --- | --- | --- | --- | --- | --- | --- | --- |
|  | **GLMM** | **LRT** | **GLMM** | **LRT** | **GLMM** | **LRT** | **GLMM** | **LRT** |
| **Sex of partner** | 0.58  (0.85) | 6.40  **0.01** | -0.78  (1.18) | 6.49  **0.01** | 2.84  (3.61) | 0.22  **0.64** | 0.22  (1.16) | 6.26  **0.01** |
| **Age at pairing** | -1.15  (0.28) | 22.69  **<0.001** | 1.08  (0.38) | 11.14  **<0.001** | 2.69  (1.26) | 4.71  **0.03** | 1.54  (0.38) | 17.97  **<0.001** |
| **Sex x Age interaction** | 0.12  (0.39) | 0.09  **0.76** | -0.15  (0.53) | 0.08  **0.77** | -1.46  (1.51) | 0.90  **0.34** | -0.63  (0.52) | 1.44  **0.23** |
| **Body length** | -0.04  (0.16) | 0.07  **0.79** | 0.11  (0.22) | 0.26  **0.61** | -0.46  (0.57) | 0.67  **0.41** | -0.06  (0.22) | 0.06  **0.81** |
| **Week of pairing**  **(seasonality)** | 0.75  (0.18) | 14.99  **<0.001** | -0.36  (0.24) | 2.16  **0.14** | -0.60  (0.67) | 0.791  **0.37** | -0.99  (0.25) | 12.07  **<0.001** |

Values given for GLMMs are model coefficients and standard errors (in brackets).

Hazard ratios for the Cox model are in italics.

Values given for LRTs are Chi-square statistics (df = 1) and P values (in bold)

NAs indicate model effects not included in analyses.
